# Metabolites-Enabled Survival of Crucian Carps Infected by *Edwardsiella tarda* in High Water Temperature

**DOI:** 10.3389/fimmu.2019.01991

**Published:** 2019-08-22

**Authors:** Ming Jiang, Zhuang-gui Chen, Jun Zheng, Bo Peng

**Affiliations:** ^1^State Key Laboratory of Bio-Control, Higher Education Mega Center, School of Life Sciences, Sun Yat-sen University, Guangzhou, China; ^2^Laboratory for Marine Biology and Biotechnology, Qingdao National Laboratory for Marine Science and Technology, Qingdao, China; ^3^Department of Pediatrics, The Third Affiliated Hospital of Sun Yat-sen University, Guangzhou, China; ^4^Faculty of Health Sciences, University of Macau, Macau, China; ^5^Southern Marine Science and Engineering Guangdong Laboratory, Zhuhai, China

**Keywords:** water temperature, bacterial infection, *Carassius carassius*, metabolome, innate immunity

## Abstract

Temperature is one of the major factors that affect the outbreak of infectious disease. Lines of evidences have shown that virulence factors can be controlled by thermo-sensors in bacterial pathogens. However, how temperature influences host's responses to the pathogen is still largely unexplored, and the study of this might pave the way to develop strategies to manage pathogenic bacterial infection. In the present study, we show that finfish *Carassius carassius*, the crucian carp that is tolerant to a wide range of temperatures, is less susceptible to bacterial infection when grown in 20°C than in 30°C. The different responses of *C. carassius* to bacterial infection could be partially explained by the distinct metabolisms under the specific temperatures: *C. carassius* shows elevated tricarboxylic acid cycle (TCA cycle) but decreased taurine and hypotaurine metabolism as well as lower biosynthesis of unsaturated fatty acids at 30°C. The decreased abundance of palmitate, threonine, and taurine represents the most characteristic metabolic feature. Consistently, exogenous palmitate, threonine, or taurine enhances the survival of *C. carassius* to bacterial infection at 30°C in a dose-dependent manner. This effect could be attributed to the inhibition on the TCA cycle by the three metabolites. This notion is further supported by the fact that low concentration of malonate, a succinate dehydrogenase inhibitor, increases the survival of *C. carassius* at 30°C as well. On the other hand, addition of the three metabolites rescued the decreased expression of pro-inflammatory cytokines including TNF-α1, TNF-α2, IL-1β1, IL-1β2, and lysozyme at 30°C. Taken together, our results revealed an unexpected relationship between temperature and metabolism that orchestrates the immune regulation against infection by bacterial pathogens. Thus, this study shed light on the modulation of finfish physiology to fight against bacterial infection through metabolism.

## Introduction

Climate is one of the most important environmental factors that influences the spread of communicable diseases prone to their epidemic (1). The outbreak of bacterial infectious disease depends on not only the pathogenicity of the bacteria but also many environmental factors, including the temperature changes. In aquaculture, infections mostly occur in spring and fall when the water temperature is between 22 and 30°C (2). The outbreak of bacterial infectious diseases depends on at least two determining factors: (1) the multiple strategies of pathogens to sense the environmental perturbations and fluctuations as cues to adjust their growth, development and pathogenesis. It is well established that the elevated temperature promotes the expression of virulence genes like type III secretion system, temperature-sensitive hemagglutinin, adhesins, and other virulence regulators in the species of *Edwardsiella, Vibrio*, and *Aeromonas* (2–6); (2) the fish's immune responses to pathogens that affected by residential water temperature. It has long been shown that the elevated temperature negatively affected fish's immune responses to vaccination and reduced the phagocytosis to bacterial pathogens (7). However, the interplay between temperature and host immune response is still largely unexplored (8, 9).

In aquaculture, infectious disease caused by bacteria is the major cause of mortalities that result in huge economic loss (10). A good research model to investigate the relationship between temperature and host immune responses might contribute to the control of infectious diseases in aquaculture and is thus highly needed. Crucian carp *Carassius auratus* is one of the most extensively cultured freshwater fish throughout the world, reaching 2.2 million tons in the year of 2010 (11). Furthermore, crucian carp is a potential model to study genome evolution and physiological adaptation as it has exhibited a striking capacity to cope with the low level of oxygen and a wide range of ambient temperatures (11, 12). The capability of crucian carp to adapt to versatile environments suggests that it is an ideal model to investigate the impacts of environmental factors on the immune responses of fish to pathogens (13).

*Edwardsiella tarda*, a zoonotic Gram-negative pathogen, infects a broad range of hosts including fish, amphibians, reptiles, and mammals, and causes edwardsiellosis (14–16). *E. tarda* represents a critical pathogen that causes huge economic and biomasses loss in aquaculture (17, 18). The outbreak of edwardsiellosis can lead to massive fish death in a short period. The infected fish shows abnormal swimming behavior, including spiral movement and floating near the water surface (19–22). Epidemiological investigation of the outbreak of *E. tarda* indicates that infection usually occurs when environmental conditions are imbalanced, such as higher water temperature, poor water quality, and high organic content (23, 24). Teleost, including crucian carps and striped bass, are ectotherm, whose body temperature is largely influenced by the water temperature. Consistently, it has different physiology or metabolism at different environmental conditions (25–28). Since metabolism is linked to immunity, it is not astonishing that bacterial infections occur more frequently at certain environmental conditions (29–32). However, the interplay among water temperature, metabolism and immunity is not well understood. The elucidation of such relationship might be useful for the development of a new strategy to fight against bacterial infection at high temperatures.

Metabolomics provides a “top-down,” integrated view of biochemistry in complex organisms that could be used to profile the metabolic response to internal and external environments. Crucial biomarkers identified from the metabolomics data could be used to reprogram the metabolome (33, 34). Antibiotic-resistant bacteria and antibiotic-sensitive bacteria have a distinct metabolome, and the crucial metabolic biomarkers can reprogram the antibiotic-resistant bacteria to become sensitive to antibiotics again, shedding light on a new strategy to manage bacterial antibiotic-resistance (33, 35–38). Similarly, tilapia showed distinct responses to *Streptococcus agalactiae* infection when grown at 20, 25, and 30°C. The metabolomics profiling of liver extract from tilapia grown at different temperatures showed that they have different metabolic responses. L-Proline, one of the crucial biomarkers, decreases along with the increasing temperatures. Interestingly, exogenous L-Proline promotes the survival of tilapias to *S. agalactiae* at 30°C (39). These results indicate that metabolic modulation is a novel approach to promote fish survival against bacterial infection at higher temperature (40–42). However, the underlying mechanism is still unknown.

In the present study, we adopted crucian carp and *E. tarda* as the research model to investigate the interplay of temperature, metabolism, and immunity. By challenging crucian carp with *E. tarda*, we found that crucian carp grown at 20°C, the optimum temperature, was more resistant to bacterial infection than those grown at 30°C. The profiling of the metabolome in fish grown at these two different environmental conditions identified three crucial biomarkers, palmitate, threonine, and taurine, which can be used to reprogram the metabolome of crucian carp and make the fish less susceptible to bacterial infection. More importantly, these three metabolites increase the expression of innate immune genes that are known to promote inflammation to kill pathogens.

## Results

### Crucian Carps Grown at Different Temperatures Show Distinct Susceptibility to *E. tarda* Infection

**Figure 1:**
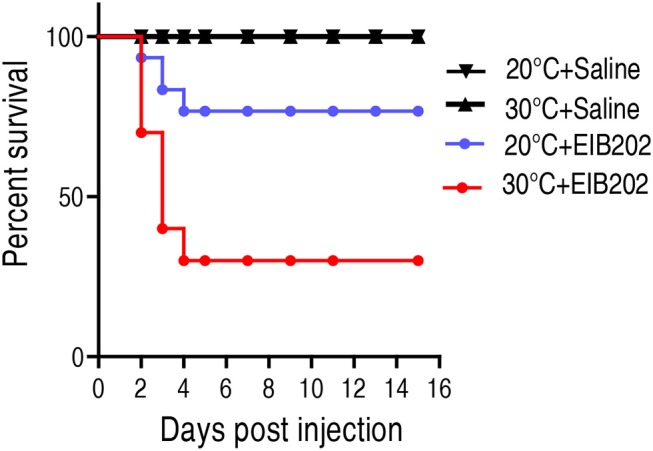
The survival of crucian carp to *E. tarda* infection cultured at 20 and 30°C. *C. carassius* (*n* = 30 per group) were acclimated at 20 or 30°C for 7 days before bacterial challenge. For bacterial infection, *C. carassius* were injected with 10 μl 1 × 10^5^ CFU/fish *E. tarda* or 10 μl saline solution as negative control. Accumulative death was monitored for a total of 15 days.

To investigate how temperature affects host's susceptibility to bacterial infection, crucian carps *Carassius carassius*, cultured at 20 or 30°C, were challenged with 1 × 10^5^ CFU *E. tarda* EIB202. Upon infection by *E. tarda*, the cumulative survival rates of *C. carassius* grown at 20°C were 76%, compared to 30% grown at 30°C (Figure 1). This result indicates that crucian carps cultured at 30°C are more susceptible to *E. tarda* infection than those cultured at 20°C.

### Water Temperature Affects Metabolomes of Crucian Carps

**Figure 2:**
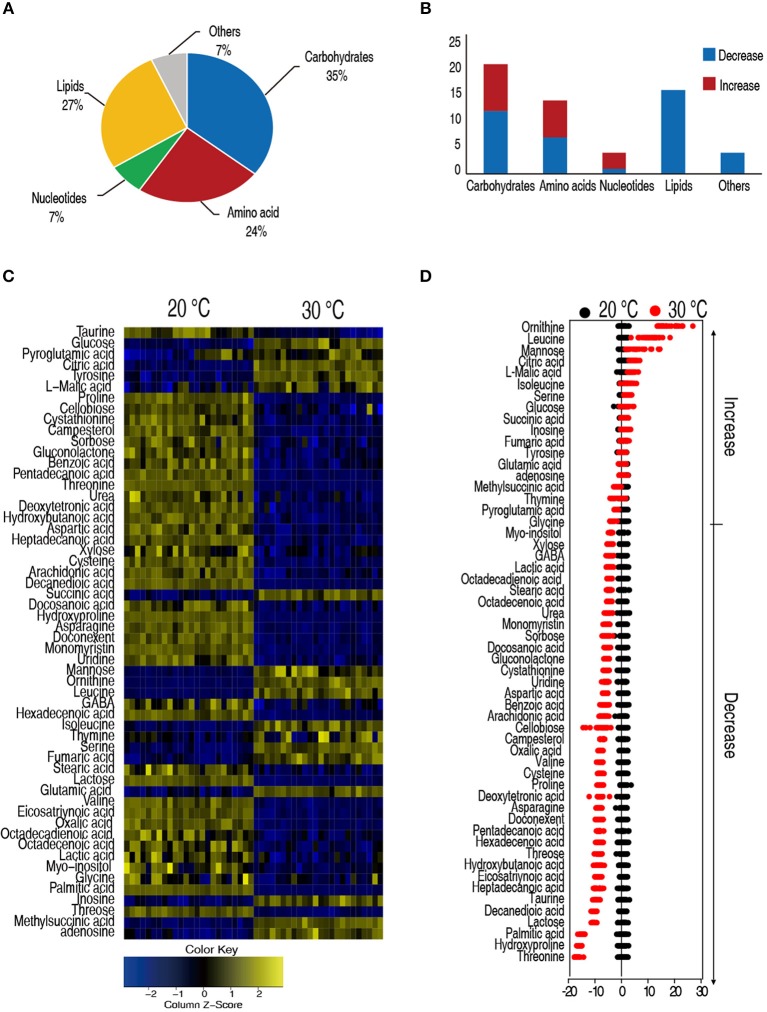
*C. carassius* cultured at 20 and 30°C had a different metabolism. **(A)** Categories of the different metabolites. Fifty six metabolites with different abundance were searched against in KEGG for their categories, and the pie chart was generated in Excel 2010 (Microsoft, USA). **(B)** The number of metabolites of different abundance in each category as shown in **(A)**. **(C)** Heat map of unsupervised hierarchical clustering of different metabolites (row). Yellow and blue indicate increase and decrease of the metabolites scaled to mean and standard deviation of row metabolite level, respectively, (see color scale) **(D)** Z scores (standard deviation from average) of metabolites identified from 30 to 20°C, which are corresponding to the data shown in **(C)**. Each point represents one technical repeat of metabolite.

Hosts adjusted their metabolism to adapt to different temperatures that have profound effects on their capability to cope with bacterial infection (39). We apply GC-MS-based metabolomics to investigate the metabolic differences between the *C. carassius* that were acclimated at 20 and 30°C for 7 days. Spleens are removed, homogenized, and the metabolites were extracted for non-targeted metabolomic analysis (Figure S1). For each spleen sample, two technical replicates were adopted. In total, 48 GC-MS data sets were generated. After data processing, 56 metabolites with different abundances were identified, followed by categorization into carbohydrates (35%), lipids (27%), amino acids (24%), nucleotides (7%), and others (7%) (Figure 2A). Among the 56 metabolites, 33 metabolites were reduced in the *C. carassius* grown at 30°C. Interestingly, the abundance of the metabolites belonging to the category of lipids were all decreased in fish cultured at 30°C (Figure 2B). A heat map of the metabolites of different abundances was generated and was shown in Figure 2C. Z-score varied between −17 and 24 at 30°C as compared to 20°C. The abundance of 17 metabolites were increased and 33 metabolites were decreased from samples in fish grown at 30°C (Figure 2D). These data suggest that *C. carassius* mounts metabolic shift when cultured at different temperatures.

### Metabolic Pathways Being Affected in *C. carassius* Cultured at 30°C

**Figure 3:**
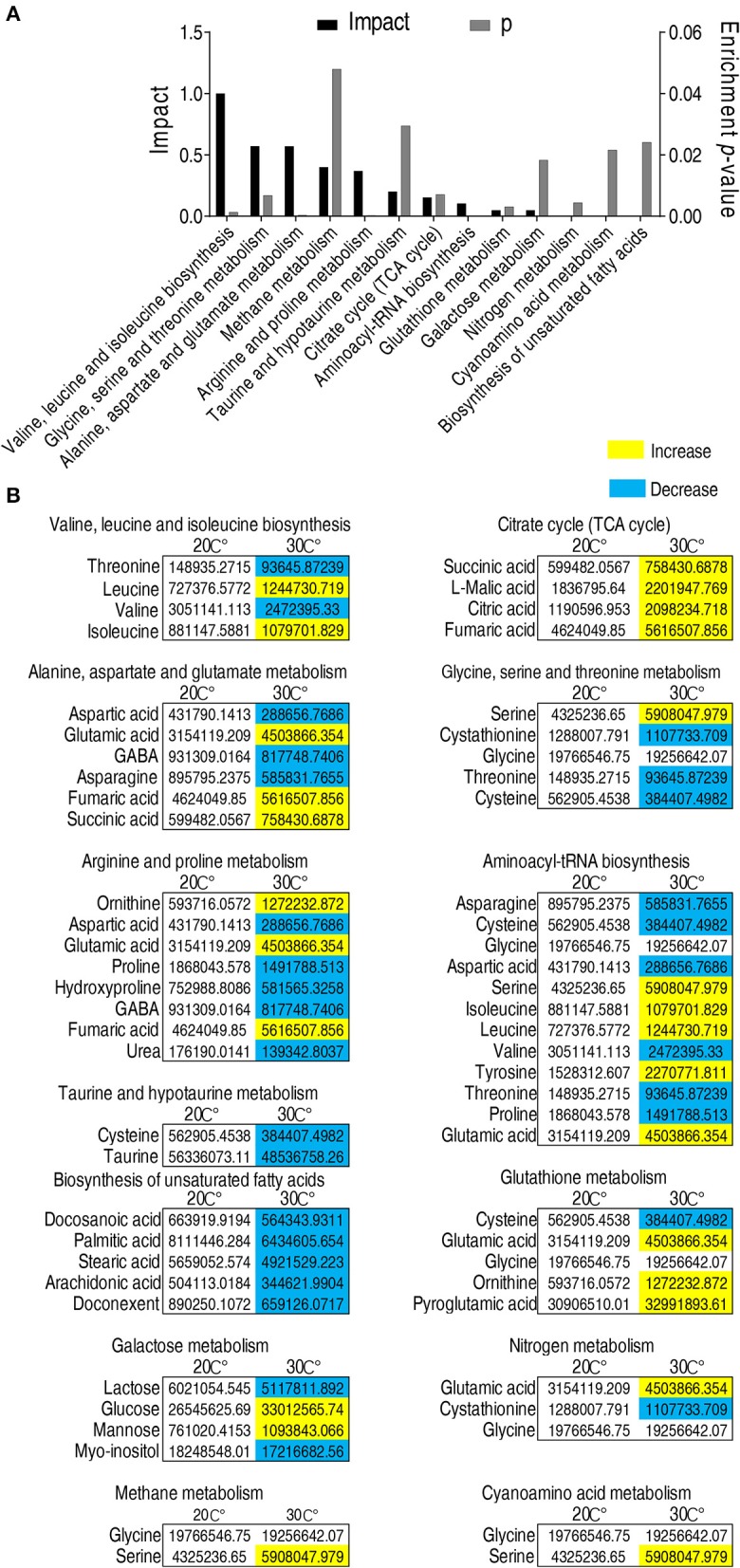
Pathway enrichment analysis of metabolites with different abundance. **(A)** Pathway enrichment analysis of different metabolites. Metabolites demonstrating different abundance were analyzed by pathway enrichment analysis using online software (http://www.metaboanalyst.ca). Significant enriched pathways are selected to plot (*p* < 0.05). **(B)** Abundance of metabolites in the enriched pathways listed in **(A)**. The metabolites in fish grown at 30°C were compared to that at 20°C. Different metabolites highlighted in yellow and blue indicate increased and decreased abundance, respectively.

Enriched metabolic pathways are crucial for the understanding of the key events during metabolic shift (43). Thus, the identified 56 metabolites with different abundance were analyzed by pathway enrichment analysis using an online software (http://www.metaboanalyst.ca). Thirteen metabolic pathways were identified. Valine, leucine, and isoleucine biosynthesis was the pathway mostly affected, followed by glycine, serine and threonine metabolism, alanine, aspartate and glutamate metabolism, methane metabolism, arginine and proline metabolism, taurine and hypotaurine metabolism, and TCA cycle. (Figure 3A). Among the 13 enriched metabolic pathways, three pathways, namely, taurine and hypotaurine metabolism, TCA cycle, and biosynthesis of unsaturated fatty acids, were of special interest because the abundance of metabolites in taurine and hypotaruine metabolism and biosynthesis of unsaturated fatty acids were all decreased, while the abundance of metabolites in the TCA cycle was increased in fish grown at 30°C (Figure 3B). These results indicate that *C. carassius* compromises taurine and hypotaurine metabolism and biosynthesis of unsaturated fatty acids but boosts the TCA cycle to cope with the temperature stress. This adaptation may make the fish more susceptible to bacterial infection.

### Taurine, Palmitic Acid, and Threonine Were the Crucial Biomarkers to Distinguish *C. carassius* Cultured at 20 or 30°C

**Figure 4:**
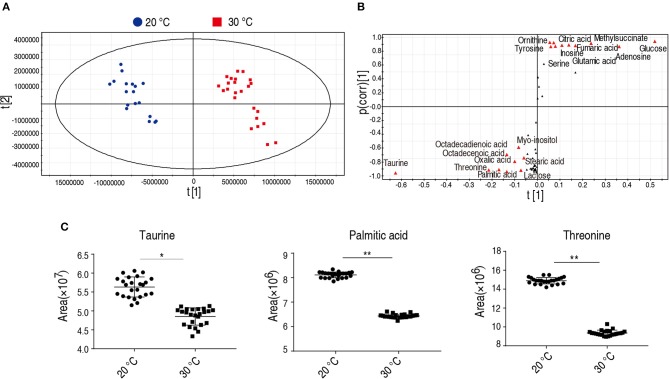
Identification of crucial biomarkers. **(A)** The PCA analysis of metabolites with difference abundance in fish grown at the 30 and 20°C to investigate intergroup difference. Each dot represents the technique replicates in the plot. The metabolites with difference abundance in fish grown at 30 and 20°C were separated by the independent factors t[1]. **(B)** S-plot, generated by OPLS-DA, to identify different metabolites of intragroup as from t[1] in **(A)**. Each triangle represents individual metabolite. The potential biomarkers are highlighted in red for metabolites whose *p* value is ≥0.05 and 0.5 for absolute value of covariance *p* and correlation *p* (corr), respectively. **(C)** Crucial biomarkers represent the metabolism of fish grown between 30 and 20°C. The abundance of the three crucial biomarkers, taurine, threonine, and palmitic acid, are compared between 20 and 30°C and presented as scatter plot. Each dot represents individual replicate. *p < 0.05; **p < 0.01.

To identify the crucial metabolites cultured at 20 and 30°C, orthogonal partial least square discriminant analysis (OPLS-DA) was conducted to recognize the sample patterns. The two groups were distributed in two quarters. Component t[1] separated 30°C group from 20°C group (Figure 4A) and Component t[1] differentiated variability within groups. Discriminating variables were shown as S-plot (Figure 4B) where cut-off values were set as grater or equal to 0.05 and 0.5 for absolute value of covariance p and correlation p(corr), respectively. Nineteen biomarkers screened by component t[1] were shown in Figure 4B (font in red). Among the 19 metabolites, the abundance of 9 metabolites were decreased in the group grown at 20°C, in which taurine, threonine, and palmitic acid showed the most significant difference. Taurine and palmitic acid are important components belonging to the taurine and hypotaurine metabolism and the biosynthesis of unsaturated fatty acids, respectively. Threonine belongs to the pathway with the second most impact (Figure 3A). Thus, taurine, palmitic acid, and threonine were the crucial biomarkers that distinguish the group cultured at 20 or 30°C. The scatter map of taurine, threonine, and palmitic acid were shown in Figure 4C.

### The TCA Cycle Was Upregulated in *C. carassius* Cultured at 30°C

**Figure 5:**
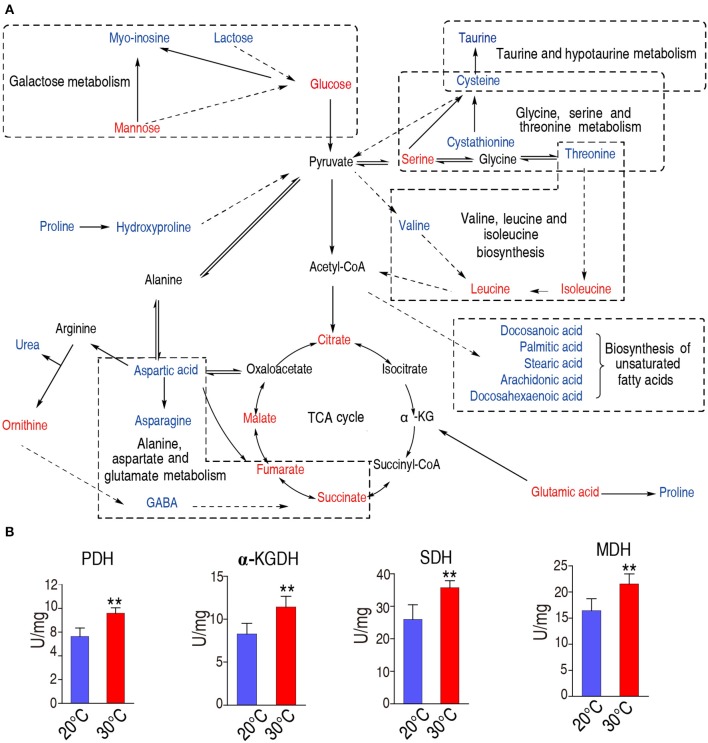
Metabolic network of metabolites of different abundance, and activity of the TCA cycle. **(A)** Integrated metabolic network in relation to different metabolites. Different metabolites are searched against KEGG, and the metabolic network was reconstructed with ChemDraw Pro 18. The red and blue stand for the increased and decreased metabolites in fish grown at 30°C, respectively. **(B)**The enzymatic activity of PDH, α-KGDH, SDH, and MDH of spleen from fish grown at 30 and 20°C were quantified. Spleens were removed, lysed and extracted for enzyme analysis, and the data were shown as histogram. The significant differences are analyzed by non-parametric Kruskal-Wallis one-way analysis with Dunn multiple comparison *post hoc* test. **p < 0.01 indicates statistic significant.

We constructed the metabolic pathways affected by the water temperature in Figure 5A, where metabolites with increased and decreased abundance are highlighted in red and blue, respectively. Our results showed that the abundances of all the identified metabolites of the TCA were increased (Figures 3B, 5A), implying that TCA cycle may play important roles in addition to taurine, threonine, and palmitic acid. To explore this possibility, we measured the activity of the enzymes of the TCA cycle, including pyruvate dehydrogenase (PDH), succinate dehydrogenase (SDH), α-ketoglutarate dehydrogenase (α-KGDH), and malic dehydrogenase (MDH). As shown in Figure 5B, the activities of PDH, SDH, α-KGDH, and MDH were higher in 30°C than those in 20°C: 7.18 ± 1.01 U/mg vs. 9.66 ± 1.34 U/mg for PDH; 25.99 ± 3.86 U/mg vs. 35.73 ± 1.86 U/mg for SDH; 8.27 ± 1.11 U/mg vs. 11.41 ± 1.14 U/mg for α-KGDH; and 16.41 ± 2.03 U/mg vs. 21.55 ± 1.69 U/mg for MDH, respectively (the percentage change and statistical significance were listed in Table 1). The increased abundance of the key metabolites in the TCA cycle, including succinate, malate, citrate, and fumarate, indicates that TCA cycle is upregulated at 30°C as to 20°C.

**Table 1:** Enzyme activity of PDH, α-KGDH, SDH, and MDH of spleen at 30 and 20°C.

**Enzyme name**	**20^**°**^C (U/mg)**	**30^**°**^C (U/mg)**	**Percentage of change (%)**
PDH	7.18 ± 1.01	9.66 ± 1.34	34.5^**^
α-KGDH	8.27 ± 1.11	11.41 ± 1.14	37.8^**^
SDH	25.99 ± 3.86	35.73 ± 1.86	37.8^**^
MDH	16.41 ± 2.03	21.55 ± 1.69	31.2^**^

*Values are represented as Means ± SEM*.

** *p < 0.01*.

### Decreased Metabolites Are Key to Survival of Fish to Bacterial Challenge at 30°C

**Figure 6:**
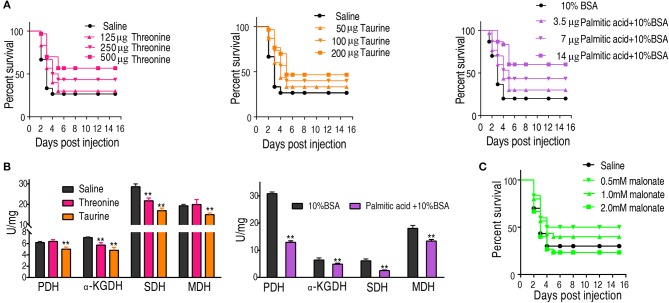
Crucial biomarkers modulate the metabolism of crucian carps and promote their survival against bacterial infections. **(A)** The survival of crucian carps in the presence of crucial biomarkers upon *E. tarda* infection. *C. carassius* was treated with saline control or different dose crucial biomarkers at 30°C for 3 days, followed by bacterial challenge through intraperitoneal injection (1 × 10^5^ CFU). The accumulative fish death was monitored for a total of 15 days' post-infection (*n* = 30 per group). **(B)** Activity of PDH, alpha-KGDH, SDH, and MDH of spleen in the presence of crucial biomarkers (200 μg taurine, 500 μg threonine, or 14 μg palmitic acid plus 10% BSA). Values are means ± SEM(*n* = 6 per group), and statistic difference is analyzed with non-parametric Kruskal-Wallis one-way analysis with Dunn multiple comparison *post hoc* test. **p < 0.01. **(C)** Percent survival of crucial carps in the presence of malonate. *C. carassius* was treated with malonate at different doses for 12 h followed by *E. tarda* challenge through intraperitoneal injection (1 × 10^5^ CFU). The accumulative fish death was monitored for a total of 15 days post-infection (*n* = 30 per group).

We hypothesized that the increase of taurine, threonine, and palmitic acid in *C. carassius* may promote fish survival of bacterial challenge at 30°C. To test this, fish was intraperitoneally injected with taurine (50, 100, and 200 μg), threonine (125, 250, and 500 μg), or palmitic acid (3.5, 7, and 14 μg), followed by the challenge with *E. tarda*. Our results showed that the three metabolites had a protective effect on bacterial infection in a dose-dependent manner (Figure 6A).

To further address the metabolic basis of how the three metabolites protect fish against bacterial infection, we measured the enzymatic activities of PDH, SDH, α-KGDH, MDH in the TCA cycle after metabolite administration. Exogenous administration of taurine and palmitic acid significantly reduced the enzymatic activity of PDH in fish (from 6.18 ± 0.25 U/mg to 5.01 ± 0.52 U/mg by taurine; from 6.41 ± 0.63 U/mg to 4.87 ± 0.52 U/mg by palmitic acid), α-KGDH (from 7.05 ± 0.17 U/mg to 4.81 ± 0.72 U/mg by taurine; from 6.09 ± 0.57 U/mg to 2.51 ± 0.31 U/mg by palmitic acid), SDH (from 28.56 ± 2.00 U/mg to 16.91 ± 1.75 U/mg by taurine; from 30.80 ± 0.52 U/mg to 12.90 ± 0.79 U/mg by palmitic acid), and MDH (from 19.29 ± 0.95 U/mg to 15.0 ± 1.15 U/mg by taurine; from 18.04 ± 0.87 U/mg to 13.38 ± 0.81 U/mg by palmitic acid) (Figure 6B; Tables 2, 3). While exogenous threonine decreased the activity of α-KGDH from 7.05 ± 0.17 to 5.73 ± 0.61, and of SDH from 28.56 ± 2.00 to 21.77 ± 1.89 U/mg, but had no effect on the activities of PDH and MDH (Figure 6B; Table 4). These results suggested that exogenous taurine and palmitic acid may downregulate TCA cycle to promote *C. carassius* survival from *E. tarda* infection at 30°C. To further validate the role of attenuated TCA cycle in fish survival, we treated *C. carassius* with a low concentration of malonate, an inhibitor of SDH, and found that fish survival was increased in 20% (Figure 6C). Therefore, the attenuation of the TCA cycle by taurine and palmitic acid, threonine or inhibitors of SDH promotes fish survival from bacterial infection at 30°C.

**Table 2:** Enzyme activity of PDH, α-KGDH, SDH, and MDH of spleen at 30°C in the presence of taurine.

**Enzyme name**	**Saline control**	**Taurine**	**Percentage of change (%)**
PDH	6.18 ± 0.25	5.01 ± 0.52	18.9^**^
α-KGDH	7.05 ± 0.17	4.81 ± 0.72	31.8^**^
SDH	28.56 ± 2.00	16.91 ± 1.75	40.7^**^
MDH	19.29 ± 0.95	15.0 ± 1.15	22.3^**^

*Values are represented as Means ± SEM*.

** *p < 0.01*.

**Table 3:** Enzyme activity of PDH, α-KGDH, SDH, and MDH of spleen at 30°C in the presence of palmitic acid.

**Enzyme name**	**10% BSA control**	**Palmitic acid + 10% BSA**	**Percentage of change (%)**
PDH	6.41 ± 0.63	4.87 ± 0.52	23.9^**^
α-KGDH	6.09 ± 0.57	2.51 ± 0.31	58.7^**^
SDH	30.80 ± 0.52	12.90 ± 0.79	58.1^**^
MDH	18.04 ± 0.87	13.38 ± 0.81	25.8^**^

*Values are represented as Means ± SEM*.

** *p < 0.01*.

**Table 4:** Enzyme activity of PDH, α-KGDH, SDH, and MDH of spleen at 30°C in the presence threonine.

**Enzyme**	**Saline control**	**Threonine**	**Percentage of change**
PDH	6.18 ± 0.25	6.38 ± 0.43	3.2^n.s.^
a-KGDH	7.05 ± 0.17	5.73 ± 0.61	18.7^**^
SDH	28.56 ± 2.00	21.77 ± 1.89	23.7^**^
MDH	19.29 ± 0.95	19.93 ± 3.43	3.3^n.s.^

*Values are represented as Means ± SEM*.

** *p < 0.01*;

n.s. *no significance*.

### Taruine and Palmitic Acid Promote the Expression of Innate Immune Genes

**Figure 7:**
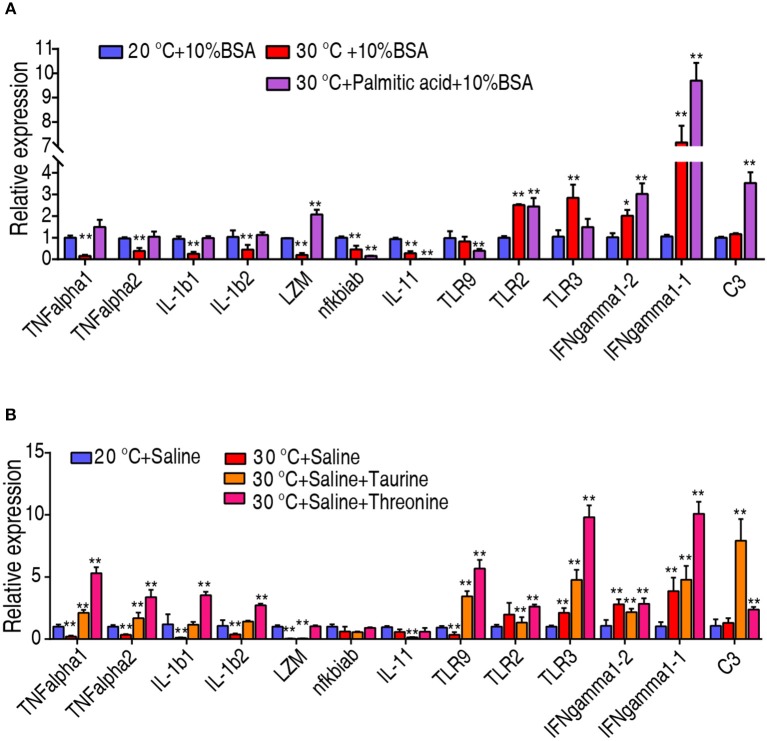
Crucial biomarkers modulate the innate immune responses of fish at 30°C. **(A,B)** qRT-PCR for cytokine genes of *C. carassius* treated with control (saline or 10% BSA) or crucial biomarkers (200 μg taurine, 500 μg threonine, or 14 μg palmitic acid plus 10% BSA) for 3 days following *E. tarda* challenge through intraperitoneal injection (1 × 10^3^ CFU). The spleens were collected 12 h post-injection for RNA extraction and qRT-PCR. Values are means ± SEM from six biological replicates. *p < 0.05; **p < 0.01.

To investigate whether the crucial biomarkers exert their effects through adjusting the fish immunity, we quantified the transcriptional level of 13 innate immune genes, including TNF-α1, TNF-α2, IL-1β1, IL-1β2, lysozyme, nfkbiab, IL-11, TLR9, TLR2, IFN-γ1-1, IFN-γ1-2, and complement component C3. Interestingly, the transcriptional level of TNF-α1, TNF-α2, IL-1β1, IL-1β2, and lysozyme were decreased when *C. carassius* was cultured at 30°C, but they can be quickly increased upon treatment with taurine, palmitic acid, and threonine (Figures 7A,B). The transcriptional expressions of TLR2, IFN-γ1-1, IFN-γ1-2, and C3 were also further boosted when the metabolites were supplemented (Figures 7A,B). Furthermore, all of these three metabolites downregulated the expression of IL-11 (Figures 7A,B). These results indicated that the three crucial biomarkers regulate innate immune response to fight against the infection at 30°C.

## Discussion

Environmental temperature affects fish host immune responses to microbial infection (44). Several endemic diseases of salmonids are more prevalent and difficult to control with the increase of water temperature (44). One reason is that the increase in water temperatures are likely to enhance the susceptibility of fish to disease and the likelihood of disease emergence (44). That temperature shift alters the immune status of *Cyprinus carpio* advanced fingerlings, while persistent sub-lethal exposure to chlorine augments temperature induced immunosuppression (26). Multiple acute temperature stress affects leucocyte populations and antibody responses in the common carp, *Cyprinus carpio L*. (27). Water temperature affects metabolism and metabolome in fish (39, 45). L-Proline, a crucial biomarker identified from temperature shift, increased the survival of tilapias infected by *Streptococcus agalactiae* in high water temperature (39). Our results further demonstrated that water temperature alters crucian carp metabolism, orchestrating innate immunity to cope with bacterial infection. Insights gained on the metabolome-mediated mechanisms may provide new therapeutic methods for the treatment of infections or preventive measures against the future outbreaks of bacterial infection.

The first core finding in the present study showed that high temperature disrupts the normal flow of the TCA cycle, making the host vulnerable to bacterial infection. The activated TCA cycle is a characteristic metabolic feature at high temperature as confirmed by the elevated activities of enzymes in pyruvate metabolism and the TCA cycle. More importantly, the inhibition of the TCA cycle promoted fish survival during bacterial infection. Therefore, the enhanced TCA cycle is critical for crucian carps to survive at higher water temperature.

The second core finding shows that the crucial biomarkers not only promote the survival of crucian carps cultured at 30°C, but also rescue the expression of innate immune genes that are repressed at 30°C. Specifically, palmitic acid, threonine, and taurine restored the expressions of TNF-α1, TNF-α2, IL-1β1, IL-1β2, and lysozyme, which were reduced at 30°C. In addition, nfkbiab, TLR9, TLR3, IL-11, and C3 were also regulated by the three metabolites. Therefore, our data provide direct evidence that these crucial metabolites, identified from water temperature-mediated metabolomes, regulate innate immune response. Among the genes of innate immunity, only 13 genes were selected as they are the only genes available for crucian carps in GeneBank, categorized to B-Jellyroll cytokines (TNF-α1 and TNF -α2), β-Trefoil cytokines (IL-1β1 and IL-1β2), Type I α helical cytokines (IL-11), Type II α helical cytokines (IFN-γ1-1 and IFN-γ1-2), TLR family (TLR 2, TLR 3, and TLR 9), lysozyme, complement system (C3), and nfkbiab (46). The function of B-Jellyroll cytokines, also called the TNF superfamily cytokines in fish, is similar to their mammalian counterparts that are expressed at early stage of bacterial infection (47). TNF-α plays a critical role in regulating inflammation by promoting phagocytosis, increasing the expression of immune genes and generation of bacterial-killing reactive oxygen species, and leukocyte homing, proliferation and migration in fish like trout (47–49). IL-1β, which belongs to the β-Trefoil cytokines, was the first interleukin identified in boy and cartilaginous fish (50). It is usually produced once the cells are activated by pattern recognition receptors (51). IL-1β plays a diverse pro-inflammatory roles that increases the number of phagocytes, phagocytic, and lysozyme activity, modulating the IL-17 family for antimicrobial activity and enhancing antibody production when co-administrated with bacterial vaccine (52–56). The TLR family are conserved cell membrane receptors that recognize pathogen-associated molecular pattern or damage-associated molecular patterns, and they function in fish species to initiate an immune response (57–59). The complement system and lysozyme are innate molecules to target bacterial cells for cell lysis by disrupting the cell membrane. They mount a non-specific immune response and represent maternal immunity from fish embryo stage, and thus are important for fish survival during bacterial infection (60). Therefore, the innate immune response was compromised by crucian carp at high temperature, but it can be rescued through metabolic reprogramming.

In addition, palmitate, threonine, and taurine protect crucian carps from *E. tarda* infection at 30°C, possibly by inhibition of the TCA cycle. More importantly, the three metabolites exhibit similar impacts on the TCA cycle and innate immune responses. These results suggest that the TCA cycle related to the innate immunity.

In summary, the present study identifies important metabolites that could promote the fish survival upon bacterial infection at 30°C, a higher water temperature than their optimum water temperature. We showed that crucian carps grown at 30°C demonstrated a metabolome that is characterized by the enhanced TCA cycle but a reduced abundance of palmitate, threonine, and taurine. Importantly, exogenous palmitate, threonine, and taurine slightly inhibit the TCA cycle and restore the altered innate immune responses repressed at the higher temperature. These results indicate that higher water temperature reduces the survival of crucian carps via metabolism, which can be reprogramed through crucial biomarkers.

## Materials and Methods

### Ethics Statement

This study was conducted in accordance with the recommendations in the Guide for the Care and Use of Laboratory Animals of the National Institutes of Health and maintained according to the standard protocols. All experiments were approved by the Institutional Animal Care and Use Committee of Sun Yat-sen University (Animal welfare Assurance Number: 16).

### Bacteria Strains and Fish

*Edwardsiedlla tarda* EIB202 is a virulent strain isolated from the outbreak of farmed turbot and is a generous gift from Dr. Yuanxin Zhang (61). EIB202 was grown in Luria-Bertani (LB) broth or 1% LB agar plate.

Crucian carp, *C. carassius*, (body length: 3 ± 0.5 cm, body weight: 2 ± 0.2 g) was obtained from Crucian Carp Breeding Corporation (Guangzhou, P.R. China). *C. carassius* was free of *Edwardsiedlla* infection through microbiological detection. *C. carassius* was normally reared in 50 L water tanks equipped with Closed Recirculating Aquaculture Systems, and the maintaining physico-chemical parameters were: dissolved oxygen: 6–7 mg/L, carbon dioxide content: <10 mg/L, pH value: 7.0–7.5, nitrogen content: 1–2 mg/L, and nitrite content: 0.1–0.3 mg/L. These animals were cultured under this condition for 2 weeks before experimental manipulation and were fed twice daily with commercial fish feed (38% crude protein, 6% crude fat, and 16% crude ash related to wet matter, 7% crude fiber and 8% moisture, based on NRC recommendations, at a ratio of 3% of body weight per day) on a 12 h/12 h rhythm of light and darkness photoperiod always. The tank was cleaned once a day by siphoning up the food debris and feces. To accommodate *C. carassius* to different temperatures, the water temperature was gradually changed until the fish were adapted to constant temperature at 20 or 30°C. The water quality was monitored regularly.

### Bacterial Challenge

To prepare *E. tarda* EIB202 for bacterial infection, a single colony was picked up from agar plate and grown in LB medium overnight. The overnight culture was diluted into fresh LB medium at 1:100 and grown with shaking at 200 rpm at 30°C until OD_600_ of the culture reached 1.0. Cells were pelleted by centrifugation and washed with 0.85% saline solution three times and suspended in saline solution.

For bacterial infection, each *C. carassius* cultured at 20 or 30°C was intraperitoneally injected with 10 μl 1 × 10^5^ CFU bacterial suspension or saline only (*n* = 30 for each treatment). These crucian carps were observed for symptoms twice daily for 15 days for accumulative death.

### Sample Preparation for GC-MS Analysis

Sample preparation was carried out as described previously (32). *C. carassius* cultured at 20 or 30°C were euthanized in ice slush (5 parts ice/1 part water, 0–4°C) for at least 10 min following cessation of gill movement, and left in the ice water for a total of 20 min after cessation of all movement to ensure death by hypoxia following the guidelines of NIH. *C. carassius* were rinsed with distilled water and then wiped thoroughly with sterilized gauze. Spleens were removed ascetically, where 25 mg of spleens were cut and immensed immediately in 1 mL cold methanol. Then, the samples were sonicated for 5 min at 10W-power setting at Ultrasonic Processor (JY92-IIDN, SCIENTZ), followed by centrifugation at 12,000 × g in 4°C for 10 min. The supernatant was collected and 10 μl 0.1 mg/ml ribitol (Sigma) was added as the internal standard. The supernatant was concentrated in a rotary vacuum centrifuge device (LABCONCO). The dried polar extracts were incubated with 80 μl methoxy amination hydrochloride (20 mg/ml pyridine) for 90 min at 37°C, followed by an addition of 80 μl N-methyl-N-trimethylsilyltrifluoroacetamide (MSTFA) (Sigma) and incubated for another 30 min at 37°C. Finally, the resulted samples were cooled down to room temperature prior to mass spectrometry analysis.

### Gas Chromatography-Mass Spectrometry (GC-MS) Analysis

GC-MS analysis was carried out with a variation on the two-stage techniques as described previously (32). In brief, 1 μL derivatized sample was injected into a DBS-MS column using splitless injection, and analysis was carried out by Agilent 7890A GC equipped with an Agilent 5975C VL MSD detector (Agilent Technologies). The initial temperature of the GC oven was held at 85°C for 5 min followed by an increase to 270°C at a rate of 15°C min^−1^ then held for 5 min. Helium was used as carrier gas and the flow was kept at 1 mL min^−1^ constantly. The MS was operated in a range of 50–600 m/z. For each sample, two technical replicates were prepared to confirm the reproducibility of the reported procedures.

### Exogenous Administration of Taurine, Threonine or Palmitic Acid and Bacterial Challenge

*C. carassius* (*n* = 330) was randomly divided into five groups, including three metabolite groups (*n* = 90 per group), and two control groups (*n* = 30 per group), and acclimatized for 7 days at 30°C. For the three metabolites groups, *C. carassius* was intraperitoneally injected with taurine (Sigma) at three doses (50, 100, or 200 μg per fish; *n* = 30 for each dose), threonine (Sigma) at three doses (125, 250, or 500 μg per fish; *n* = 30 for each dose), or palmitic acid (Sigma) at three doses (3.5, 7.0, or 14.0 μg per fish; *n* = 30 for each dose). For the two control groups, they were either injected with saline only (*n* = 30) as a control to taurine and threonine groups, or with 10% bovine serum albumin (Sigma) as a control to palmitic acid group. The injection was conducted once daily for 3 days. Afterwards, *C. carassius* was challenged by intraperitoneal injection of *E. tarda* EIB202 with a dose of 10 μl 1 × 10^5^ CFU/fish, and the fish mortality was observed for 15 days for accumulative death.

To quantify the transcriptional level of innate immune genes by qRT-PCR, *C. carassius* was intraperitoneally injected with taurine (200 μg per fish; *n* = 20), threonine (500 μg per fish; *n* = 20), or palmitic acid (14 μg per fish; *n* = 20) once daily for 3 days. Then, *C. carassius* were challenged with *E. tarda* EIB202 with a dose of 10 μl 1 × 10^3^ CFU per fish. The spleens were aseptically removed and collected 12 h post-infection as stated above for RNA isolation.

### Measurement of Enzyme Activity

Enzyme activities were measured as described previously (37, 62). In brief, spleens freshly removed from fish were rinsed with pre-cooled 1 × PBS, resuspended in lysate buffer, and disrupted by sonication for 5 mins at 10W power setting. Following centrifugation, the supernatant, defined as the total protein, was transferred to a new tube, and the protein concentration in the supernatant was determined by Bradford assay. The total proteins of 100 μg were applied for pyruvate dehydrogenase or α-ketoglutarate dehydrogenase assay, and 250 μg of total proteins for succinate dehydrogenase or malate dehydrogenase assays. The enzymatic assay was conducted by mixing an equal volume of the protein sample and reaction buffer to a final volume of 200 μL in 96-well plate. The plate was further incubated at 37°C for 15 min, and the absorbance was read at 570 nm on a microplate reader. The enzyme activity was calculated by plotting against the standard curve. The reaction mixtures are as follows: PDH reaction mixture: 0.5 mM 3-(4,5-di methyl thiazol-2-yl)-2,5-diphenyltetrazolium bromide (MTT) (Sangon Biotech), 1 mM MgCl_2_ (Sigma), 6.5 mM phenazine methosulfate (PMS) (Sangon Biotech), 0.2 mM thiamine pyrophosphate (TPP) (Sangon Biotech), and 2 mM sodium pyruvate (Sigma) and 50 mM phosphate buffered saline (PBS) (Invitrogen); KGDH reaction mixture: 0.5 mM MTT, 1 mM MgCl_2_, 6.5 mM PMS, 0.2 mM TPP, 2 mM α-ketoglutaric acid sodium salt (Sigma) and 50 mM PBS; SDH reaction mixture: 0.5 mM MTT, 13 mM PMS, 5 mM sodium succinate (Sigma), and 50 mM PBS; MDH reaction mixture: 0.5 mM MTT, 13 mM PMS, 50 mM malic acid disodium salt (Sigma), and 50 mM PBS.

### RNA Isolation and qRT-PCR

Total RNA was isolated with Trizol (Invitrogen, USA) and quantified by Nanodrop (Thermo Scientific). Reverse transcription-PCR (qRT-PCR) was carried out using Prime-Script^TM^ RT reagent Kit with gDNA eraser (Takara, Japan) with 1 μg of total RNA according to manufacturer's instructions. The experiment was performed in six biological replicates.

qRT-PCR was performed with a LightCycler-480 (Roche, Germany) using SYBR Premix Ex Taq II Kits (Takara, Japan) following the manufacture's instruction. The experiment was performed in six biological replicates. Primers for each gene were listed in Table 5, and each primer pair was specific. *Actin, tubulin and GAPDH* genes were chosen as the internal control. The relative expression of each gene was determined by comparative threshold cycle method (2^−ΔΔ*CT*^ method).

**Table 5:** Primers used for QRT-PCR analysis.

**Gene**	**Primer**	**Sequence (5^**′**^-3^**′**^)**
Actin	Forward	gggatgggacagaaggacag
	Reverse	acgcagctcgttgtagaagg
Tubulin	Forward	ctgctgggaactctattgtc
	Reverse	ctccaggtctacaaacacag
GAPDH	Forward	tgacccctccagtatgacca
	Reverse	gagggcctcctcaataccaa
TNFalpha1	Forward	tcacgctcaacaagtctcag
	Reverse	tggtcctttctccagtaaag
TNFalpha2	Forward	ccgctgtctgcttcacatt
	Reverse	ggccttggaagtgacattt
IL-1b1	Forward	atgcgctgctcaacttcat
	Reverse	ctggcccttattttgttgag
IL-1b2	Forward	caaagcgatcctcttcattt
	Reverse	attcgggtcatcagttttaa
IL-11	Forward	ttcgagtggctgaacagaac
	Reverse	aggcccagtcacagaagagc
TLR-2	Forward	cttagatgggctcactcatc
	Reverse	gggtgggagacatctttaag
TLR-3	Forward	tagatgccagctacaactcttt
	Reverse	ggctccccaattaacttcag
TLR-9	Forward	gccaacccatgttatcagtc
	Reverse	ggtgtcgcagatttttaaga
IFNgamma1-1	Forward	ctacgggtcctgaaagactt
	Reverse	gcctgggaagtagttttctc
IFNgamma1-2	Forward	tctggggagtatgcttgttga
	Reverse	gcctgggaagtagttttcttg
nfkbiab	Forward	cagtttggcgcagacatt
	Reverse	gcgcctttgctgattagaag
LZM	Forward	tgtgtctgatgtggctgtgc
	Reverse	tgcacacatagttgccaagtga
C3	Forward	tggggatggatctgaaaca
	Reverse	tgcccatgatgaggtacga

### Data Processing and Statistical Analysis

For the GC-MS data analysis, metabolites from the GC-MS spectra were identified by searching against National Institute of Standards and Technology (NIST) library used the software of NIST MS search 2.0. The resulting data matrix was normalized using the concentrations of added internal standards, which were subsequently removed so that the data could be used for modeling consisted of the extracted compound. Peak areas of all identified metabolites were normalized by ribitol as internal standard. Statistical difference of metabolites in each sample was obtained by Kruskal–Wallis test and Mann–Whitney test using SPSS 23.0 (IBM Corp, USA). Z-score and hierarchical clustering were used to analyze the normalization area. Hierarchical clustering was completed in the R platform (https://cran.r-project.org). Multivariate statistical analysis included principal component analysis (PCA) and orthogonal partial least square discriminant analysis (OPLS-DA) implemented with SIMCA 12.0 (Umetrics, Umeå, Sweden).

For the data analysis of qRT-PCR and enzyme activity, non-parametric Kruskal-Wallis one-way analysis with Dunn multiple comparison *post hoc* test was used, SPSS 23.0, *p* < 0.05 was considered significant.

## Data Availability

All datasets generated for this study are included in the manuscript and/or the Supplementary Files.

## Ethics Statement

This study was conducted in accordance with the recommendations in the Guide for the Care and Use of Laboratory Animals of the National Institutes of Health and maintained according to the standard protocols. All experiments were approved by the Institutional Animal Care and Use Committee of Sun Yat-sen University (Animal welfare Assurance Number: 16).

## Author Contributions

BP conceptualized and designed the project and wrote the manuscript. MJ and ZC performed the experiments. MJ and JZ performed the data analysis. BP, MJ, ZC, and JZ interpreted the data. All the authors reviewed the manuscript.

### Conflict of Interest Statement

The authors declare that the research was conducted in the absence of any commercial or financial relationships that could be construed as a potential conflict of interest.
