# Supplementary material for: Metabolites-Enabled Survival of Crucian Carps Infected by Edwardsiella tarda in High Water Temperature
Source: Front Immunol. 2019 Aug 22;10:1991. doi: 10.3389/fimmu.2019.01991 (PMC6713922; doi:10.3389/fimmu.2019.01991)
Supplement: Supplementary file 1 [file Data_Sheet_1.docx]

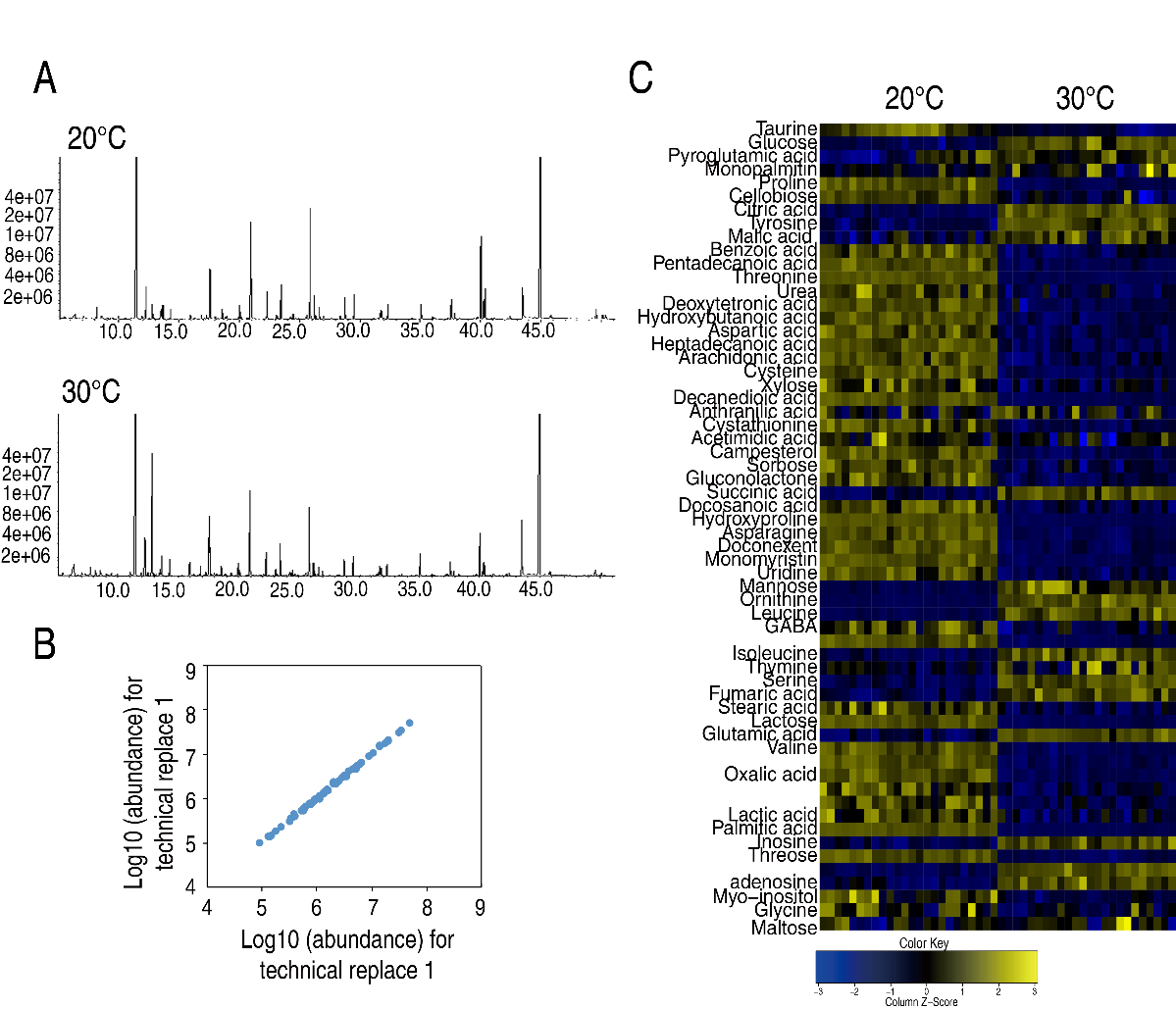


**Figure S1: Metabolic profile of crucian carps cultured at 30 °C and 20 °C.** (A) the outputs of GC-MS （B）The reproducibility of the data (C) The heatmap of GC-MS data of crucian carp cultured at 30 °C and 20 °C
